# Insoluble Vascular Amyloid Deposits Trigger Disruption of the Neurovascular Unit in Alzheimer’s Disease Brains

**DOI:** 10.3390/ijms22073654

**Published:** 2021-04-01

**Authors:** Luis O. Soto-Rojas, B. Berenice Campa-Córdoba, Charles R. Harrington, Andrés Salas-Casas, Mario Hernandes-Alejandro, Ignacio Villanueva-Fierro, Marely Bravo-Muñoz, Linda Garcés-Ramírez, Fidel De La Cruz-López, Miguel Ángel Ontiveros-Torres, Goar Gevorkian, Mar Pacheco-Herrero, José Luna-Muñoz

**Affiliations:** 1 Facultad de Estudios Superiores Iztacala, Universidad Nacional Autónoma de México, México City 54090, Mexico; oskarsoto123@unam.mx; 2 Departamento de Fisiología, Escuela Nacional de Ciencias Biológicas, Instituto Politécnico Nacional, México City 07738, Mexico; berecordoba21@gmail.com (B.B.C.-C.); adnil_gr@yahoo.com.mx (L.G.-R.); flacruz90@hotmail.com (F.D.L.C.-L.); 3 National Dementia BioBank, Ciencias Biológicas, Facultad de Estudios Superiores Cuautitlán, Universidad Nacional Autónoma de México, México City 53150, Mexico; marelybravo@hotmail.com; 4 School of Medicine, Medical Sciences and Nutrition, University of Aberdeen, Aberdeen AB25 2ZD, UK; c.harrington@abdn.ac.uk; 5 Instituto de Ciencias de la Salud, Área Académica de Gerontología Universidad Autónoma del Estado de Hidalgo, Hidalgo 42060, Mexico; andres_salas15@yahoo.com.mx; 6 Departamento de Bioingeniería, Unidad Profesional Interdisciplinaria de Biotecnología del Instituto Politécnico Nacional (UPIBI-IPN), México City 07340, Mexico; mhernandes@ipn.mx; 7 CIIDIR, Durango, Instituto Politécnico Nacional, Durango, Becario COFAA, Durango City 34220, Mexico; ifierro62@yahoo.com; 8 Tecnologico de Monterrey, Escuela de Ingeniería y Ciencias, Toluca, Estado de México 50110, Mexico; miguelontiveros@tec.mx; 9 Instituto de Investigaciones Biomédicas, Universidad Nacional Autónoma de México (UNAM), México City 70228, Mexico; gokar@unam.mx; 10 Neuroscience Research Laboratory, Faculty of Health Sciences, Pontificia Universidad Catolica Madre y Maestra, Santiago de los Caballeros 51000, Dominican Republic; 11 Banco Nacional de Cerebros-UNPHU, Universidad Nacional Pedro Henríquez Ureña, Santo Domingo 2796, Dominican Republic

**Keywords:** Alzheimer’s disease, fibrillar amyloid, pyroglutamate-modified amyloid-beta peptides, neurovascular unit, blood–brain barrier, caspase-5

## Abstract

Alzheimer’s disease (AD) is a neurodegenerative disease, characterized histopathologically by intra-neuronal tau-related lesions and by the accumulation of amyloid β-peptide (Aβ) in the brain parenchyma and around cerebral blood vessels. According to the vascular hypothesis of AD, an alteration in the neurovascular unit (NVU) could lead to Aβ vascular accumulation and promote neuronal dysfunction, accelerating neurodegeneration and dementia. To date, the effects of insoluble vascular Aβ deposits on the NVU and the blood–brain barrier (BBB) are unknown. In this study, we analyze different Aβ species and their association with the cells that make up the NVU. We evaluated post-mortem AD brain tissue. Multiple immunofluorescence assays were performed against different species of Aβ and the main elements that constitute the NVU. Our results showed that there are insoluble vascular deposits of both full-length and truncated Aβ species. Besides, insoluble aggregates are associated with a decrease in the phenotype of the cellular components that constitute the NVU and with BBB disruption. This approach could help identify new therapeutic targets against key molecules and receptors in the NVU that can prevent the accumulation of vascular fibrillar Aβ in AD.

## 1. Introduction

Alzheimer’s disease (AD) is a progressive neurodegenerative disease characterized by memory impairment and cognitive and functional decline. Neuropathological hallmarks of AD include the intra-neuronal tau-related lesions and the accumulation of amyloid β-peptide (Aβ) in the brain parenchyma, in the form of neuritic plaques (NPs), and cerebral blood vessels as cerebral amyloid angiopathy (CAA). Furthermore, the disruption of the neurovascular unit (NVU) has been linked to AD and other neurodegenerative diseases [1].

The two-hit vascular hypothesis of AD states that vascular risk factors (*hit 1*) lead to the dysfunction of the blood–brain barrier (BBB) and the NVU, initiating a cascade of events that precedes dementia. The cerebrovascular damage reduces Aβ clearance at the BBB and increases its production by cleaving amyloid-β precursor protein (APP), leading to Aβ accumulation (*hit 2*) [2].

The main Aβ variants detected in the human brain are Aβ1-40 and Aβ1-42. However, a significant proportion of Aβ in AD brain consists also of N-terminally truncated species [3]. AβN3(pE) and AβN11(pE), Aβ peptides, including N-terminal pyroglutamate at positions 3 and 11, have been demonstrated to be a major N-terminal truncated constituent of intracellular and extracellular deposits in AD brain [4,5,6]. Diffuse amyloid plaques, one of the earliest forms of amyloid deposits, have also been shown to contain AβN3(pE) [7]. Pyroglutamate-containing Aβ species show an increased aggregation propensity and have been proposed to play an important role during the initiation of the disease, even before the onset of clinical symptoms [4]. It has been proposed that Aβ molecules tend to aggregate and form oligomers, protofibrils, and finally mature fibrils, triggering neuronal dysfunction (directly affecting the synapse or indirectly promoting glial activation) [8,9,10]. In the last decade, the oligomer hypothesis was postulated, in which it is suggested that Aβ oligomers may initiate the cascade of neuropathological events in AD [11]. The Aβ oligomers are soluble and may spread throughout the brain, being considered the most toxic Aβ form [11]. Amyloid fibrils are larger and insoluble, and assemble into NPs, being recognized by thiazine red (TR) [12,13,14].

Alterations in the NVU compromises the brain microcirculation and vascular neuroinflammatory responses [15]. The NVU, or the minimal functional unit of the brain, includes vascular cells, glial cells, neurons, and endothelial cells [16,17,18], with the latter present in the BBB. The BBB is a specialized structure in the cerebral vasculature, which limits the entry of toxic agents, pathogens, and cells into the brain[19]. It has been postulated that deposition of Aβ in the vessels can alter the NVU and promote or exacerbate AD by triggering several pathological events, such as neuroinflammation, chronic hypoperfusion, and ischemia [20,21,22]. However, the species of Aβ that are deposited on the walls of the cerebral vessels have not been fully elucidated.

Thus, this work aimed to demonstrate the association between different Aβ species and NVU disruption in sporadic AD brains. Our results demonstrate that the amyloid peptides Aβ1-40 and AβN3 (pE) are the major species that are deposited as fibrils on the walls of blood vessels. Interestingly, these insoluble deposits are related to a phenotypic loss of pericytes, vessel-associated microglia, and astrocytic end-foot processes, as well as with the breakdown of tight junctions.

## 2. Results

### 2.1. Distinct Deposition of Aβ Species in Blood Vessel Walls of AD Brains

Different species of Aβ have been described in AD brains [23]. To analyze the forms of Aβ that are present in the walls of the cerebral vessels, we carried out immunofluorescent staining for the full-length Aβ species (Aβ1-40 and Aβ1-42), the N-truncated/modified Aβ species (AβN3 [24] and AβN11 [pE]), and for fibrillar, thiazin red (TR)-positive Aβ deposits.

**Figure 1:**
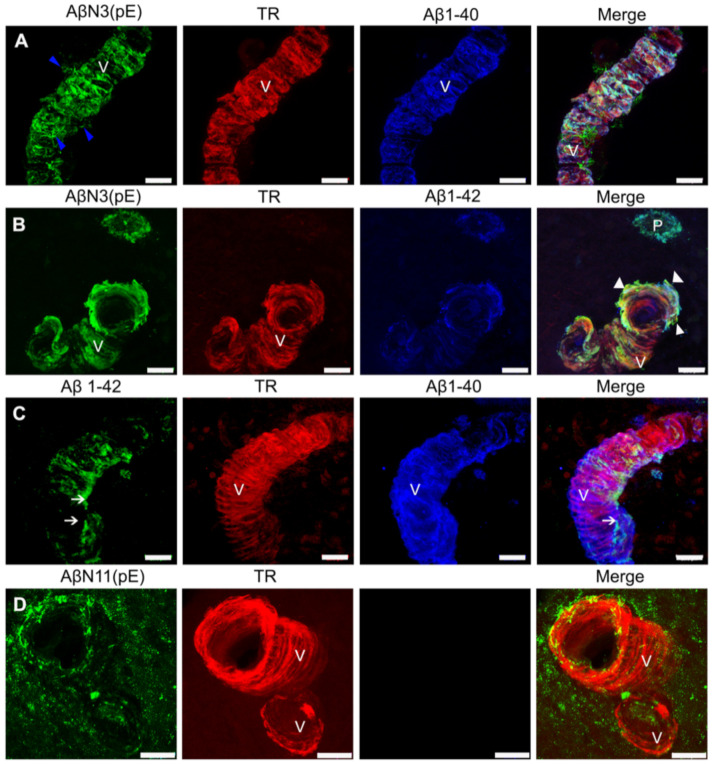
Double and triple immunofluorescence analysis of Alzheimer’s disease (AD) brains for the simultaneous detection of fibrillar Aβ species on blood vessel walls (V). (**A**) Representative micrograph sections of insoluble (stained with thiazine red (TR); red channel), AβN3(pE) peptide (green channel) and Aβ1-40 (blue) deposits that span the entire vessel wall, with blue arrowheads pointing to suspected string vessels. (**B**) Triple immunostaining against AβN3(pE) peptide (green), TR (red), and Aβ1-42 (blue); the amyloid deposits are observed both in the amyloid plaque (P) and in the wall of the blood vessel (V), and, for the latter, structural alterations are observed (arrowhead). (**C**) Representative micrograph of dense, insoluble (TR; red channel) Aβ1-40 (blue channel) deposits and patchy deposits of Aβ1-42 (green channel, white arrows), on the blood-vessel wall. (**D**) Representative micrograph of dense, insoluble amyloid deposits (TR; red channel) and diffuse perivascular and blood vessel wall deposits of AβN11(pE) (green channel). The scale bar = 20 μm is common for all micrographs.

All the Aβ forms studied were found in blood vessel walls (Figure 1). Interestingly, the aggregation pattern showed distinct patterns of reactivity. While AβN3 [pE] and Aβ1-40 deposits were found along the entire vessel wall (Figure 1A–C), Aβ1-42 immunoreactivity showed a patchy distribution along the vessel axis (Figure 1C). A diffuse immunoreactivity for truncated peptide AβN11 [pE] was observed around the vessel (Figure 1D). String vessels (Figure 1A, blue arrowheads) and structural alterations (Figure 1B, white arrowheads) positive only for AβN3 [pE] were detected in the blood vessels in AD brains.

### 2.2. Extracellular, Vascular, and Intracellular Amyloid Deposits Trigger Morphological Changes in Microglia

**Figure 2:**
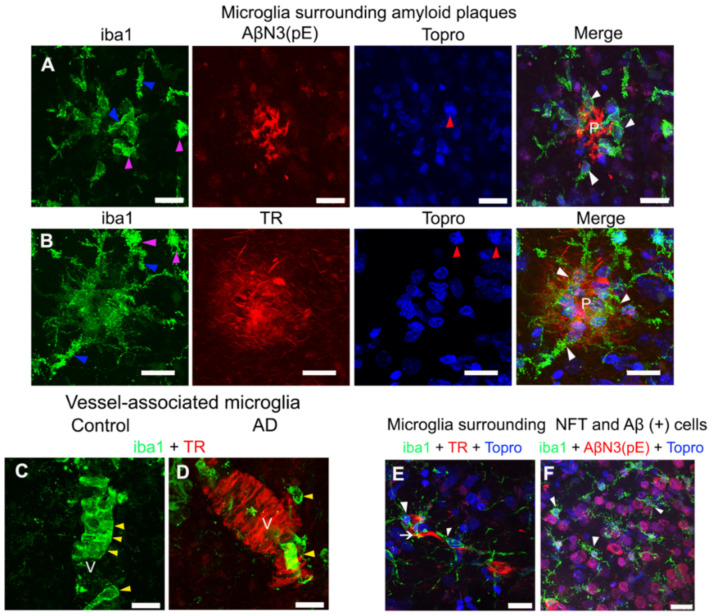
Representative micrographs of double and triple immunostainings of microglia in AD brains. (**A**) Microglia (green channel) surrounding (white arrowheads) an amyloid plaque (P) with AβN3(pE) peptide deposits (red channel). (**B**) Microglia (green channel) surrounding (white arrowheads) a fibrillar TR-positive amyloid plaque (P; red channel). Changes in the morphology (purple arrowheads) of microglia ramifications (blue arrowheads) and the nuclear morphology (blue channel; red arrowheads) are indicated in both panels. Control (**C**) and AD (**D**) brains show vessel (V)-associated microglia (yellow arrowheads; green channel) counterstained with TR (red channel). (**E**,**F**) Representative micrographs of microglia (white arrowheads; green channels) surrounding a neurofibrillary tangle (NFT) (white arrow; red channel) and Aβ (+) cells (red channel), respectively. Some tissues were counterstained with TO-PRO^®^-3 (blue channel). The scale bar = 20 μm.

Proliferation and activation of microglia in the brain are prominent AD features [25]. We observed activated microglia closely associated with amyloid plaques (Figure 2A,B; white arrowheads) in AD. These microglia were found around AβN3 [pE] plaque and fibrillar TR-positive plaque. Microglia exhibited an intimate interaction of their processes with Aβ. Microglia showed a thickening of their processes (Figure 2A,B; blue arrowheads), to develop an amoeboid-like morphology (Figure 2A,B; purple arrowheads). Nuclear condensation (Figure 2B; red arrowheads) was observed by using TO-PRO^®^-3 dye.

On the other hand, vessel-associated microglia (Figure 2C,D), showed a different distribution pattern in control and AD brains. In control brains (Figure 2C), continuity was observed in the microglia cells along the blood vessel (Figure 2C; yellow arrowheads). In AD (Figure 2D), an apparent decrease in immunostaining for microglia was noticed. Furthermore, these microglia did not show continuity throughout the TR-positive vessel (Figure 2D; yellow arrowheads).

Interestingly, microglial cells were also found to be associated with TR-positive neurofibrillary tangles (NFTs) (Figure 2E; white arrowheads). In this case, microglia exhibited a resting morphology. Dystrophic microglia were found around Aβ-positive-cells (Figure 2F; white arrowheads). Soluble oligomers of AβN3 [pE] were found intracellularly.

### 2.3. Fibrillar Amyloid Deposits in the Cerebral Vasculature Trigger Morphological Changes in Astrocytic End-Foot Processes

**Figure 3:**
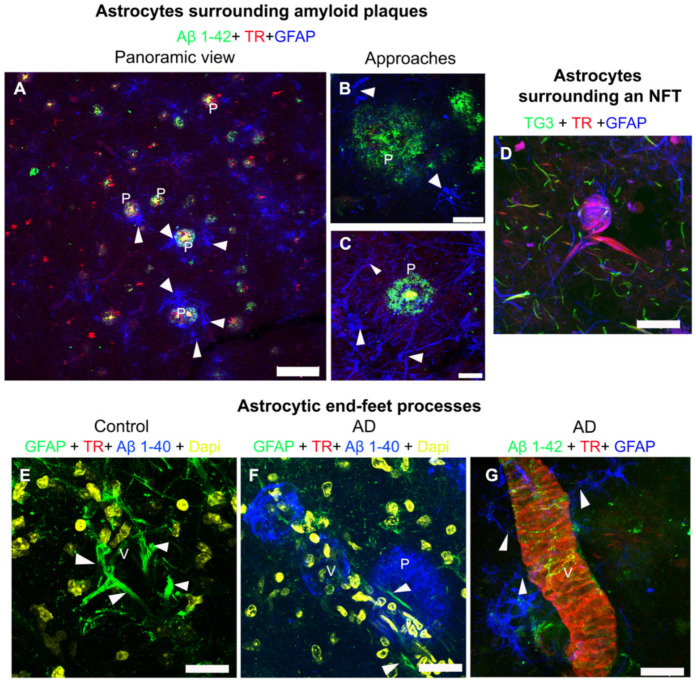
Representative micrographs of triple and quadruple immunostainings for astrocytes in AD brains. (**A**–**C**) Representative images of triple immunostaining against Aβ1-42 (green channels), TR (red channels), and glial fibrillary acidic protein (GFAP) (blue channels), showing how the astrocytes surround (white arrowheads) the amyloid plaques (P), both in a panoramic view (**A**) and in closer detail (**B**,**C**). (**D**) Representative micrograph of an NFT that was labeled, using TG3 (green channel) and TR (red channel), surrounded by astrocytes (blue channel). (**E**–**G**) Astrocytic end-foot processes (white arrowheads) around the blood vessels (V) are shown. (**E**) Control brain and (**F**) AD brain, after quadruple immunostaining for GFAP (green channels), TR (red channels), Aβ1-40 (blue channels), and DAPI (4′,6-diamidino-2-phenylindole) nuclear counterstain (yellow channels). (**G**) Triple immunostaining against Aβ1-42 (green channel), TR (red channel), and GFAP (blue channel). The scale bars = 100 µm for the panoramic view (a) and 20 μm for others.

Previous studies have shown the presence of astrocytes surrounding NPs [26]. Consistently, we observed astrocytes surrounding the NPs (Figure 3A–C). Interestingly, we observed a greater level of astrocyte associated with Aβ1-42 fibrillar amyloid plaques than with diffuse plaques (Figure 3A–C, white arrows).

Importantly, astrocytes in the vicinity of the TR-positive NFTs were noticed (Figure 3D). Some of these astrocytes appeared intermixed with the tau filaments that make up the NFTs. Moreover, TG3-positive tau was seen in the vicinity of the NFTs. In Figure 3E–G, astrocytic end-foot processes in AD and control brains are shown. These end-foot processes were thicker and more abundant in control brains (white arrowheads; Figure 3E), compared to AD brains (white arrowheads; Figure 3F,G). Astrocytic end-foot processes appeared much thinner when associated with insoluble vascular amyloid deposits (Figure 3G, white arrowheads).

### 2.4. The α-Smooth Muscle Actin Is Fundamentally Associated with Soluble Aβ Deposits in AD

Pericytes are a component of the NVU and play a crucial role in regulating cerebral blood flow when they contract [27]. Several studies have demonstrated that most capillary pericytes in the central nervous system contained little or no α -SMA expression, a key protein for their contraction [28,29,30]. Thus, in order to analyze the possible damage of the microvasculature, we investigated α-SMA reactivity.

**Figure 4:**
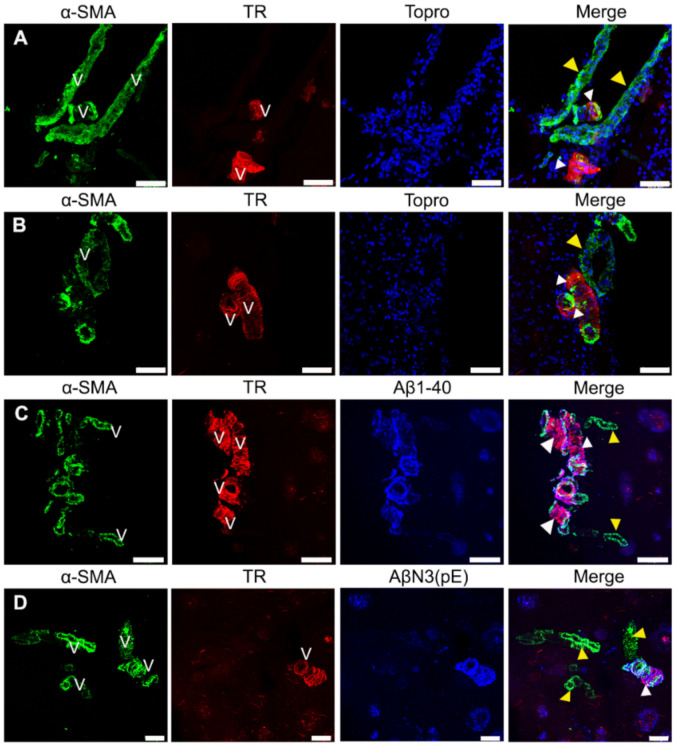
Representative micrographs of triple immunostainings for α-SMA-positive pericytes in AD brains. (**A**,**B**) Immunostaining for pericytes (green channels), TR (red channels), and contrast with TO-PRO^®^-3 (blue channels). (**C**,**D**) Immunostaining for pericytes (green channels), TR (red channels), and deposits of Aβ1-40 and AβN3(pE) (blue channels). Extensive (yellow arrowheads) and occasional (white arrowheads) immunoreactivity for pericytes along the blood vessel (V) is observed in all panels. The scale bars = 60 µm for (**A**,**B**), 100 μm for (**C**), and 40 μm for (**D**).

We found expression of α-SMA in AD (Figure 4) but not in control brains (data not shown). The α-SMA immunoreactivity was observed throughout the cerebral vasculature (Figure 4A–D). Interestingly, expression of α-SMA was greater in those areas that were devoid of Aβ immunoreactivity (Figure 4A–D; yellow arrowheads) than insoluble deposits (Figure 4A–D; white arrowheads).

### 2.5. Disruption of the Tight-Junction Integrity in the Cerebral Vessels in AD Brains

Previous studies have suggested significant BBB alterations in both vascular and degenerative forms of dementia. Tight junctions (TJs) are major components of the BBB that physically obstruct the inter-endothelial space and restrict blood-borne substances from the peripheral circulation to the CNS [31]. Claudin-5 (Cl-5) is one of the proteins involved in TJs. It is expected that the “sealing role” of TJs and Cl-5 is altered in pathological situations. Thus, we performed immunofluorescent staining with antibodies against Cl-5, to analyze the integrity of TJs and brain microvasculature.

**Figure 5:**
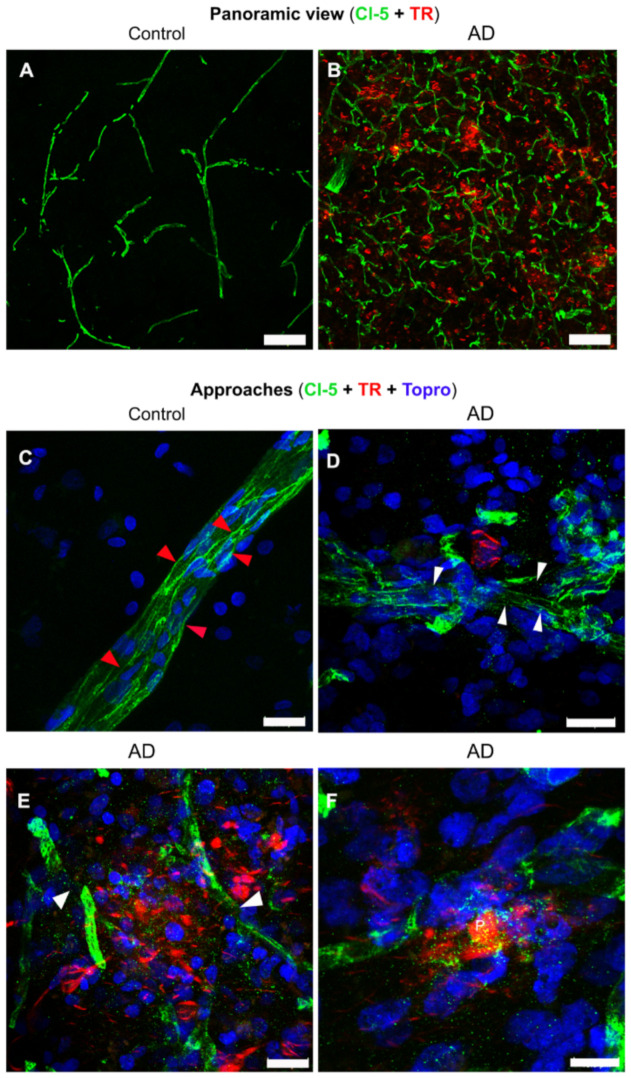
Double and triple immunofluorescent staining of tight junctions in AD and control brains. Representative panoramic views of control (**A**) and AD (**B**) brains with double immunostaining for claudin- 5 (Cl-5; green channels) and TR (red channels). (**C**–**F**) Representative images of triple immunostaining in control (**C**) and AD (**D**–**F**) brains, for Cl-5 (green channels), TR (red channels), and TO-PRO^®^-3 (blue channels). Continuity (red arrowheads) and discontinuity (white arrowheads) of the tight junctions are indicated. The scale bars = 100 µm for the panoramic views (**A**,**B**) and 20 μm for magnifications (**C**–**F**).

We observed a notable increase in the expression of Cl-5 in AD, compared to the control brains (Figure 5A,B). Cl-5 presented a characteristic expression of continuity and well-defined borders in the microvasculature of control brains (Figure 5C, red arrowheads). However, Cl-5 showed a fragmented pattern with undefined edges (Figure 5D,E, white arrowheads) in AD. Moreover, Cl-5 remnants (dotted pattern) were observed in the vicinity of the fibrillar amyloid plaques (Figure 5E,F), due to disruption of TJs.

### 2.6. Activated Caspase-5 Is Expressed in Blood Vessels of AD Brains

**Figure 6:**
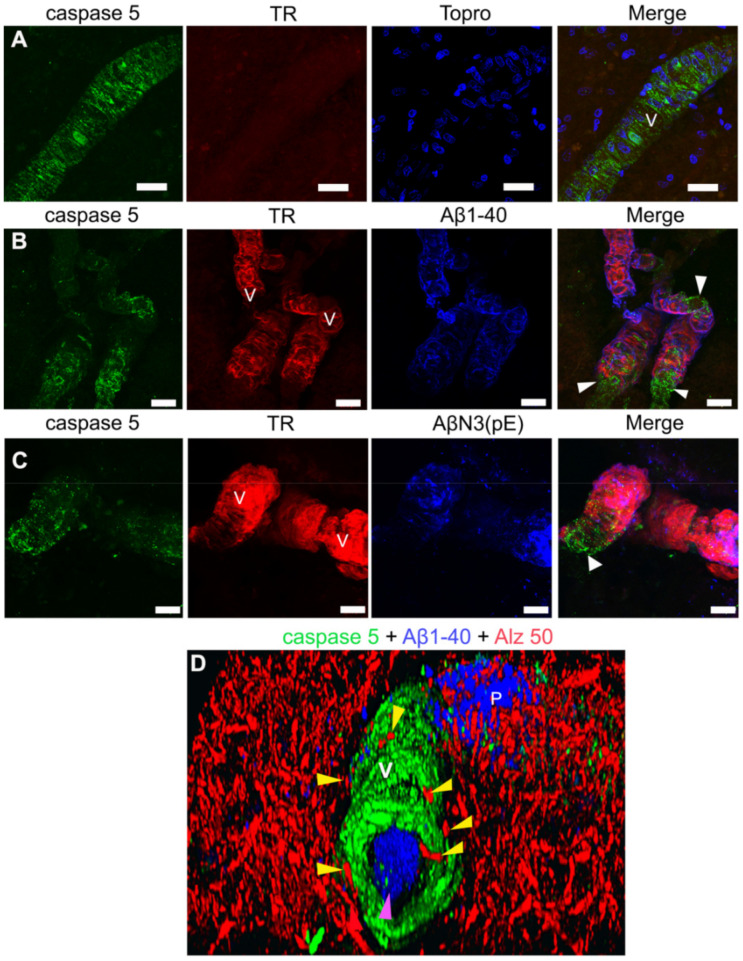
Representative micrographs of caspase-5 immunoreactivity in the blood vessels (V) of AD brains. (**A**) Immunostaining for caspase-5 (green channel), TR (red channel), and TO-PRO^®^-3 (blue channel). (**B**,**C**) Triple immunostaining against caspase-5 (green channel) TR (red channel), Aβ1-40 (panel B; blue channel), and AβN3(pE) peptide (**C**) (blue channel). (**D**) Three-dimensional (3D) reconstruction from several stacks of laser scanning confocal microscopy images of a blood vessel (V) immunoreactive for caspase-5 (green channel), surrounding pathological tau (red channel; yellow arrowheads), and Aβ1-40 deposits (blue channel) on a plaque (P), as in the lumen of the blood vessel (purple arrowhead). The soluble Aβ vascular deposits are associated with extensive immunoreactivity for caspase-5 (white arrowheads). The scale bar = 20 μm is common for all micrographs.

In AD, an inflammatory environment surrounds the cerebral microvasculature, favored by the accumulation of Aβ, and associated with NVU dysfunction [32]. Caspase-5 is an enzyme belonging to the family of inflammatory caspases [33], which, under physiological conditions, is expressed at very low levels in the brain [34]. We observed the presence of caspase-5 in AD brains (Figure 6). Specifically, caspase-5 was present extensively in the cerebral vasculature in AD (Figure 6), but was not present in healthy brains (data not shown). To our knowledge, we are the first group to report the presence of this caspase in the AD brain. Interestingly, caspase-5 was expressed in greater levels in those areas that were devoid of Aβ immunoreactivity in the blood vessel (Figure 6B,C; white arrowheads), as compared with insoluble amyloid deposits (Figure 6A–C). We also observed that amyloid deposits were also present in the vessel lumen (Figure 6D; purple arrowhead) and in pathological tau surrounding the cerebral vasculature (Figure 6D; yellow arrowheads) in AD brains.

## 3. Discussion

Neuropathological hallmarks of AD are the NFTs, the accumulation of Aβ in the brain parenchyma and around cerebral blood vessels (cerebral amyloid angiopathy; CAA), and NVU dysfunction [1]. The NVU consists of different cell types, including vascular cells such as (1) brain endothelial cells lining the cerebral vascular tree, pericytes covering microvascular capillaries, and vascular smooth muscle cells (VSMCs) enwrapping cerebral arterioles and arteries; (2) glial cells, such as vessel-associated microglia, astrocytic end-foot processes, and oligodendrocytes; and (3) neurons. The NVU includes the different cell types involved in the formation of the BBB [1,16,17,24]. CAA is almost universally found in AD patients [35]. However, the specific Aβ species associated with blood vessels have not been fully elucidated. In this work, we analyzed Aβ1-40, Aβ1-42, and N-truncated peptides like AβN3 (pE) and AβN11 (pE) deposition in the wall of blood vessels. These peptides are cytotoxic and the principal markers in AD.

We observed extensive immunoreactivity of different fibrillar Aβ species along the walls of the blood vessels, being more evident for the Aβ1-40 and AβN3(pE) peptide species. These vascular amyloid deposits are associated with structural vascular alterations and phenotypic loss of pericytes, vessel-associated microglia, and astrocytic end-foot processes. Interestingly, we observed in AD brains a loss in continuity of the TJs that constitutes the BBB, as well as extensive caspase-5 immunoreactivity in the cerebral vasculature.

The presence of AβN3(pE) deposits in the cerebral vasculature has been described in several animal models of AD. However, their presence was not detected in brains with sporadic AD [5] and transgenic mouse models [36,37]. The cerebrovascular deposits of AβN3(pE) have been reported preferentially in familial AD [38]. However, we found extensive fibrillar/insoluble vascular deposits of both truncated Aβ species and the full-length Aβ species (Aβ1-40) in several sporadic AD cases. Recently, it has been proposed that the balance of Aβ maturation in CAA and plaques defines distinct pathological subgroups of Aβ amyloidosis, inclusive of AβN3(pE) [39].

**Figure 7:**
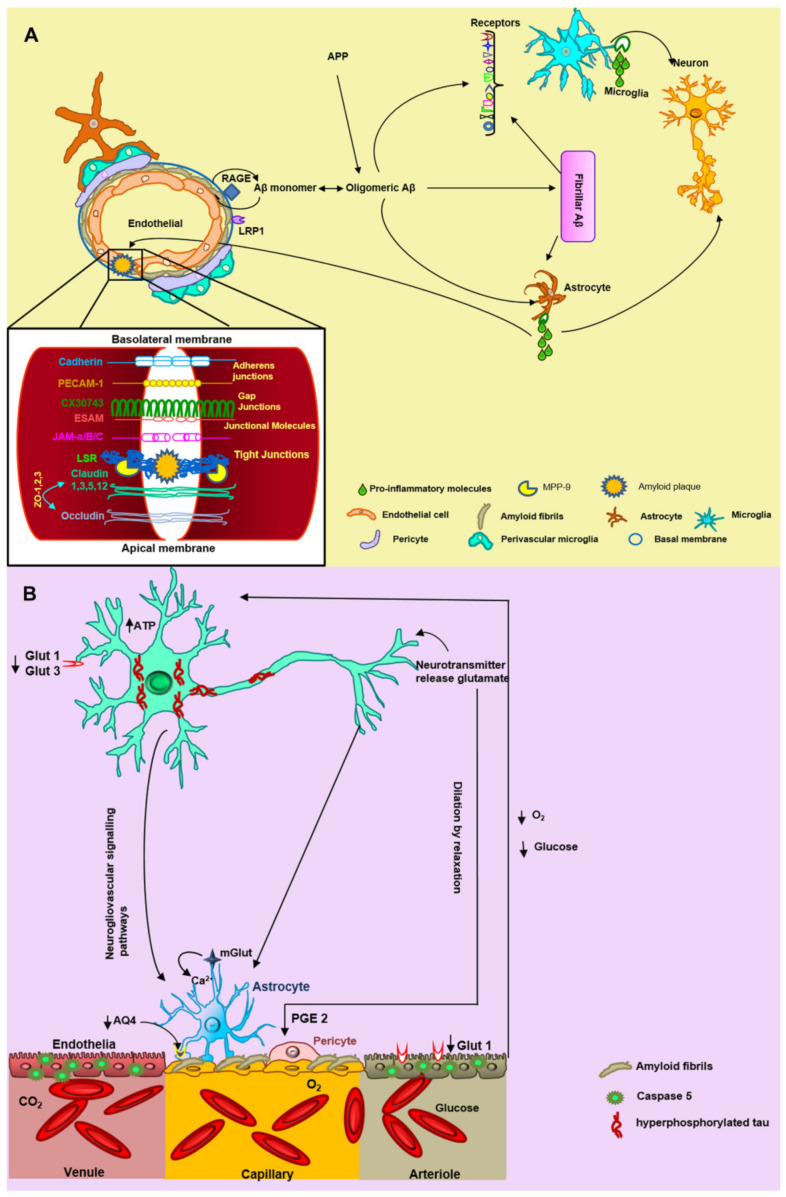
The hypothesis of neurovascular unit (NVU) alteration triggered by Aβ fibrillary deposits. (**A**) Aβ monomers are incorporated into oligomers and fibrils in the brain, triggering NVU degeneration. Some endothelial receptors such as the receptor for advanced glycation end-products (RAGE) and low-density lipoprotein receptor-related protein 1 (LRP1) are involved in the vascular Aβ accumulation and thus with blood–brain barrier (BBB) damage. The microglial receptors TLR2, MARCO, SCARA ½, CR3 (β2 integrin), CR4, SCARB2, CD36, TLR4-TLR6, CD47, α6β1 integrin, SR-A1, and TREM 2 interact with fibrillar Aβ. Subsequently, microglia and astrocytes secrete pro-inflammatory molecules that promote neuronal and vascular damage. Fibrillar Aβ aggregates or NP could trigger the breaking of tight junctions specifically by metalloproteinase 9 (MMP9). (**B**) There is an accumulation of fibrillar Aβ in endothelial cells causing oligemia (decrease in oxygen and glucose levels). In the neuron, there are two important glucose receptors GLUT 1 and GLUT 3, which are decreased during Alzheimer’s disease, leading to phosphorylation of tau, excitotoxicity, and cell death. Various studies have found a decrease in aquaporin 4 (AQ4) in astrocytes, generating edema of the astrocytic feet. The pericytes undergo dilation by relaxation due to the presence of PGE 2. The neurotransmitter glutamate is released from the neuron to the astrocyte, where it produces an increase in intracellular calcium. We rely on the genome.jp/keg and expasy.org platforms and the results obtained in this study for the proposed hypothesis.

Cerebrovascular amyloid accumulation could disrupt the NVU in AD patients and exacerbate pathology [40]. Atrophy of pericytes, swelling of the astrocytic end-foot processes, loss of perivascular plexus, decrease in vascular smooth muscle actin, and accumulation of laminin in basement membranes have all been observed in AD brains [41]. Accordingly, we detected a loss of the cellular NVU components, but this event was associated with the vascular amyloid fibrillar deposits. Nevertheless, the mechanisms by which fibrillar Aβ could aggregate in the vasculature and go on to cause NVU damage in AD are not yet clear. First, it has been proposed that microvascular amyloid seeds could recruit wild-type Aβ peptides and promote their assembly into amyloid fibrils, in a prion-like way, triggering cytotoxicity [22]. Second, the altered expression of some molecules and receptors (RAGE and LRP1 receptors at the BBB; Aβ chaperone proteins, such as apoE and apoJ; and possibly vascular-specific genes, such as mesenchyme homeobox gene 2 (MEOX2) and myocardin (MYOCD)) is associated with control of Aβ efflux and influx of the brain[1]. The dysfunction of these molecules (Figure 7) leads to a decrease of brain perfusion, due to a compromised interstitial fluid (ISF) drainage, resulting in a cerebral blood flow (CBF) reduction by vascular Aβ accumulation [42]. This oligemia (Figure 7) is reflected in a loss of oxygen and glucose, causing excitotoxicity and plasticity impairment, and in an increase in anaerobic brain metabolism [43]. Therefore, oligemia could alter the pH, electrolyte balances, and water gradients, leading to the development of edema, white matter lesions, and glutamate accumulation. Likewise, it could also trigger a loss of neuronal and BBB glucose receptors (GLUT1 and GLUT3, respectively) that can lead to tau phosphorylation [41], probably through the mitogen-activated protein kinase pathway [44] (Figure 7). Conversely, tau deposition is generally not a prominent feature of CAA pathology, but it has been observed around Aβ-laden vessels in sporadic and hereditary CAA [45]. It has been suggested that tau could induce leukocyte trafficking into the brain in vivo in a transgenic animal model and functional damage to brain endothelial cells in an in vitro BBB model [41]. In this study, we observed pathological tau surrounding the cerebral vessels, which could trigger the previously mentioned effects. All of these events could contribute to alteration in the NVU and neuronal death [40]. We also observed different effects associated with oligemia and amyloid angiopathy, such as string vessels, vascular structural change, irregularities in the capillary surface, and BBB discontinuity. String vessels may form as a result of endothelial cell death, followed by the collapse of the capillary walls, leaving only remnants of the extracellular matrix caused by a decrease of CBF; they are not functional in maintaining BBB integrity [42]. It has been suggested that Aβ accumulates substantially along string vessels [46]. In support of this hypothesis, we observed that string vessels are associated with fibrillar Aβ vascular deposits. Interestingly, we only observed this event with the vascular deposits of the AβN3(pE) peptide.

Several studies have reported that focal vascular regression and decreased microvascular density occur in AD [47,48]. On the other hand, the low levels of MEOX2 specifically found in brain endothelial cells isolated from AD patients have been shown to mediate aberrant angiogenic responses of human brain endothelium to angiogenic factors, such as vascular endothelial growth factor (VEGF), both in vivo and in vitro, leading to premature capillary pruning [48]. This effect was associated with areas close to fibrillar amyloid plaques. Hence, we noticed that fibrillar amyloid plaques were also related to BBB discontinuity and also presumptively with its breakdown (Figure 7). It has been demonstrated that the exposure to oligomeric forms of Aβ significantly decrease levels of occludin, Cl-5, and ZO-1, and compromises BBB integrity [49,50]. The decrease in TJ proteins might be partly explained by the vascular-associated matrix metalloproteinase-9 (MMP-9; Figure 7) activity, because TJ proteins and basement membrane are substrates for this enzyme [51]. Future experiments are necessary to determine if diffuse or fibrillar amyloid plaques are likely to trigger BBB rupture, in addition to vascular amyloid deposits.

On the other hand, the oligomeric theory has gained interest in recent years, where it has been postulated that Aβ oligomers, rather than fibrils, can cause several cellular toxic effects and trigger neuronal death [11]. This hypothesis has been supported by the findings from a number of studies. It has been shown that Aβ oligomers can cause damage and increased permeability of the neuronal lipid bilayer, alter calcium homeostasis, and favor the neuroinflammatory response, all of which are events not associated with Aβ fibrils [52,53,54,55,56]. In this study, we observed a pathological effect of Aβ fibrillar on the cells that constitute the NVU. However, as the NVU cells present several receptors for the Aβ oligomers [57], it is possible that these could trigger the first toxic events with insoluble amyloid deposits leading to the later degeneration of these cells in AD brains.

Nevertheless, the mechanisms by which fibrillar Aβ could bind to cells of the NVU and consequently cause degeneration are not yet clear. Exposure of microglia to fibrillary Aβ in vitro leads to activation and production of chemokines, cytotoxic cytokines [58] and reactive oxygen species (ROS)[59,60], which could lead to neuronal toxicity and degeneration [61] (Figure 7). It has been suggested that, in healthy brains, microglial cells are in “resting” state, characterized by small-bodied cells with long, finely branched processes. In contrast, under pathological conditions, microglia are in an “active” amoeboid shape in which they have begun to swell and retract their processes, or may have completely lost them. Interestingly, we found that microglial cells surrounding amyloid plaques have amoeboid-like morphology but also exhibit nuclear condensation suggesting that they are entering an apoptotic process. We also observed that the microglia that surround the NFT have a resting morphology, while those that surround the cells with intracellular deposits of Aβ have an amoeboid shape, suggesting that intracellular Aβ deposits may be associated with the activation of these cells.

It is believed that microglia, the major phagocytic cells in the brain, are part of the first step in degrading soluble and fibrillar Aβ through Scavenger Receptors (SRs) [62]. There are many of these receptors that trigger activation of signaling cascades and these are depicted in the schematic in Figure 7.

Caspases are cysteine-dependent aspartate-directed proteases. Caspases are fundamentally involved in apoptosis and inflammation. Previous studies support the notion that caspase-mediated cleavage of critical proteins contributes to neurodegeneration in AD [63]. Caspases could trigger Aβ levels during apoptosis. Furthermore, Aβ could initiate apoptosis, which in turn would increase caspase activity. Some caspases have been described in AD. Expression of caspase-3 has been reported in AD cases, where it is co-localized with NFTs and NPs in the brain [64]. Activated caspase-3 immunoreactivity was also found to be present in a large subset of neurons, blood vessels, and glial cells. Caspase-6 [63] has been described in degenerating neurons and neurites of AD but not in control brains [63]. In this study, for the first time, we describe the expression of activated caspase-5 throughout the cerebral vasculature of AD brains. Caspase-5 was not evident in the brains from healthy individuals. It has been shown that caspase-5 has been related to apoptosis, inflammation, proliferation, and that its expression, activation and catalytic activity are inducible by proinflammatory agents, such as IL-1β, TNF-α, and LPS in Human Retinal Pigment Epithelial Cells [65,66]. Future studies will be required to determine the role of caspase-5 in cerebral vessels and its specific relationship with Aβ and NFTs in AD.

As with vessel-associated microglia, astrocytic end-foot processes could limit the disease in the early stages. As the amyloid vascular pathology progresses, it could trigger molecular alterations and even degeneration. It has been suggested that astrocytes can bind to fibrillar Aβ via CD36, CD47, RAGE, ApoE, α7nAChR, and glycoprotein receptors. Although the most common pathway is through RAGE and the NF-kB pathway, increased expression of TNF- α, IL-1 β, COX2, and other cytokines [67] may also promote a neuroinflammatory environment (Figure 7). As a consequence, the BBB structure and expression/distribution of tight junctions are altered [68]. Moreover, in the temporal cortex of AD patients, reduced mRNA expression of the water channel aquaporin 4 (AQP4; Figure 7) and activated astrocyte marker glial fibrillary acidic protein (GFAP) in association with the severity of CAA pathology [69] and in AD-related pathology, including Aβ burden and Braak stage [70]. In our study, we noticed that vascular amyloid deposits altered the end-foot processes morphology. We observed that the amyloid fibrillar deposits caused thinning of these terminations, in comparison with the diffuse Aβ deposits or the control brains. These events could reflect cellular degeneration and death. The astrocytic end- foot dysfunction during AD may exacerbate Aβ accumulation by disturbing cerebrovascular Aβ clearance, along with the ISF drainage via the glymphatic systems [71], influencing arteriolar tone by a steady low-level efflux of prostaglandin-E2 (PGE2) as a result of basal [Ca^2+^] intracellular fluctuations [72]. This is consistent with an assumption that astrocyte dysfunction is key to the breakdown of neurovascular coupling [40].

Both astrocytes and microglia could promote endothelial autophagy of the endothelial cells by activation of AKT and PI3K signaling [73]. Additionally, it has been described that high concentrations of Aβ induce mitochondria dysfunction, DNA fragmentation, and notorious endothelial cell death [74]. For instance, it has been suggested that Aβ-induced apoptosis in vascular cells via death receptors (DRs). DR4 and DR5, as key players in CAA cell dysfunction [75], could trigger mitochondrial dysfunction and activation of caspase 8, and then caspase 3, resulting in protein cleavage and further apoptosis (Pereira et al. 2004).

Finally, it has been proposed that degeneration of pericytes could be a pathophysiological key to NVU dysfunction in AD [76,77,78]. In post-mortem studies, an accumulation of blood-derived proteins (including fibrinogen, thrombin, plasminogen, immunoglobulin G, and albumin) has been found in the hippocampus and cortex of AD subjects, and this is associated with pericyte degeneration [77]. Pericyte degeneration in AD may further exacerbate parenchymal and vascular Aβ accumulation [78]. Furthermore, degenerating pericytes could trigger BBB breakdown, CBF reductions, and hypoxia, which, in turn, initiates age-dependent secondary neuronal and synaptic changes associated with neuronal and synaptic dysfunction [76]. In this study, we observed strong immunoreactivity of contractile pericytes in AD brains.

## 4. Materials and Methods

### 4.1. Brain Tissue

Human brain tissue was obtained from the National Dementia BioBank, Mexico, following the institutional bioethics guidelines. Tissues from six sporadic AD (age range 65 to 90 years, disease duration 10–15 years, and 3–6 h postmortem delay) and six cognitively normal patients (age range 65 to 90 years, and 3–6 h postmortem delay) were used. The diagnosis of AD followed the National Institute of Neurological and Communicative Disorders and Stroke—Alzheimer’s Disease and Related Disorders Association (NINCDS-ADRDA)criteria [79]. AD tissue with stages IV–VI (according to Braak’s staging system) was used. Braak IV–VI is associated with a high degree of CAA [39]. Control cases were defined by the absence of amyloid plaques, including cases with primary age-related tauopathy and CAA [39].

### 4.2. Immunofluorescent Staining

Blocks of temporal cortex from AD (*n* = 6) and control (*n* = 6) brains were fixed by immersion in a solution of 4% paraformaldehyde in phosphate-buffered saline (PBS), pH 7.4, at 4 °C. Sections of 50 μm in thickness were cut on a sliding microtome (Jung Histoslide 2000R; Leica, Heidelberg, Germany). Antibody epitopes were retrieved, following treatment in citrate buffer (0.1 M citric acid, 0.1 M sodium citrate, pH 6.0) at 100 °C, for 15 min. Tissues designated to assess Aβ were incubated with 70% formic acid, for 20 min, at room temperature. Non-specific sites were blocked with 0.2% IgG-free albumin (Sigma-Aldrich, St. Louis, MO, USA) in PBS, for 1 h, at room temperature. Tissues were then incubated with the primary antibodies cocktail (Table 1), overnight, at 4 °C, and then with the secondary antibodies for 1 h (Table 2). Sections were counterstained with thiazine red (TR), to identify beta-pleated sheet conformation [12], and with TO-PRO^®^-3 or DAPI for staining nuclei. Slices were mounted in Vectashield antifade mounting medium (Vector Laboratories, Burlingame, CA, USA).

### 4.3. Immunofluorescent Staining for Tight Junctions

Fresh-frozen temporal cortex tissues were obtained from AD (*n* = 3) and control (*n* = 3) individuals. Tissue was cryopreserved in OCT (optimal cutting temperature) compound (Sakura Finetek, Torrance, CA, USA), and 50-μm sections were cut, using a cryostat (Leica, Nussloch, Germany). Slices were fixed with paraformaldehyde, at room temperature, for 20 min, and permeabilized with 100% methanol, at −20 °C, for 20 min. From this step, a brain-slice chamber system maintained the brain-slice preparations in a constantly humid environment. Non-specific sites were blocked with 1% bovine serum albumin (BSA) (Sigma-Aldrich, St. Louis, MO, USA), in PBS, for 1 h, at room temperature. Lipofuscin autofluorescence was removed with Sudan B Black (SBB) solution (0.1% SBB in 70% ethanol) for 10 min. Tissues were incubated with the rabbit polyclonal anti claudin-5 antibody (1:300; Abcam; Cambridge, UK), overnight, at 4 °C, and then with FITC-tagged goat–anti-rabbit IgG (Jackson ImmunoResearch Laboratories, Inc., West Grove, PA) for 1 h, at room temperature. Sections were counterstained with TO-PRO^®^-3 or DAPI. Slices were mounted in Vectashield antifade mounting medium (Vector Laboratories).

### 4.4. Confocal Microscopy

Double and triple-immunolabeled sections were examined with a confocal laser scanning microscope (TCS-SP8, Leica, Heidelberg, Germany), using 20×, 40×, and a 100× oil-immersion plan Apochromatic objective (NA 1.4). Ten to fifteen consecutive single sections were sequentially and simultaneously scanned at 0.8–1.0 µm intervals, for two or three channels throughout the *z*-axis of the sample. The collection of images was projected and analyzed onto the two-dimensional plane, using pseudo-color display of green (FITC), red (TRITC), and blue (CY5). Fluorochromes were excited at 488 nm (for FITC), 540 nm (for TRITC), and 650 nm (for CY5).

## 5. Conclusions

Our results suggest that, in the late stage of AD, there are extensive vascular deposits of fibrillar Aβ, containing both full-length and truncated Aβ species. Furthermore, these insoluble vascular amyloid deposits were associated with a loss of cellular elements that constitute the NVU and in a disruption of the BBB. The high caspase-5 immunoreactivity found in the cerebral vasculature suggests that there is an inflammatory response or pyroptosis of cells that constitute the NVU. Therefore, further research must focus on elucidating the pathological mechanisms involved in NVU dysfunction in AD brains, including the role of different Aβ species and caspase-5. The functionality of the NVU should be considered as a therapeutic target for AD.

## Figures and Tables

**Table 1:** List of primary antibodies used in this study.

Primary Antibody	Protein and Epitope	Species; Isotype	Dilution	Reference
AβN3(pE)	Aβ peptide bearing N-terminal pyroglutamate at position 3	Mo. and Rb.	1:100	[6]
AβN11(pE)	Aβ peptide bearing N-terminal pyroglutamate at position 11	Rb.	1:100	[6]
Aβ1-40	Synthetic Aβ1-40; N-terminal epitope within amino acid residues 1–12	Mo.	1:300	Sigma-Aldrich
Aβ1-42	Synthetic Aβ1-42; C-terminal epitope within amino acids 36–42	Rb.	1:200	Thermo-Scientific
Polyclonal anti α smooth muscle actin (α-SMA)	N-terminus region of human alpha smooth muscle actin	Rb.	1:500	GeneTex
Ionized calcium-binding adapter molecule 1 (Iba1)	Synthetic peptide within human Iba1 aa 1–100 (Cysteine residue).	Rb.	1:1000	Wako
GFAP	Glial fibrillary acidic protein	Rb and Mo.	1:500	Thermo-Scientific
ALZ50	Amino acids: 5–15, 312–322 Structural conformational change in tau	Mo; IgM.	1:20	[80]
TG-3	Regional conformational change in tau dependent on phosphorylation at Thr231 and Ser235	Mo.; IgM	1:40	[81]
Caspase-5	Activated caspase-5 resulting from cleavage adjacent to Ser331	Rb.	1:500	GeneTex

Mo = mouse; Rb = rabbit.

**Table 2:** List of secondary antibodies used in this study.

Secondary Antibody	Isotype
Alexa488	Goat anti-MoIgG (γ) Goat anti-MoIgM (µ) Goat anti-RbIgG (H+L)
Alexa546	Goat anti-MoIgG (γ) Goat anti-MoIgM (µ) Goat anti-RbIgG (H+L)
CY5	Goat anti-MoIgG (γ) Goat anti-MoIgM (µ) Goat anti-RbIgG (H+L)

## Data Availability

The data that support the findings of this study are available from the corresponding author upon reasonable request.
